# A case of pancreatic ductal adenocarcinoma growing within the pancreatic duct mimicking an intraductal tubulopapillary neoplasm

**DOI:** 10.1007/s12328-025-02098-y

**Published:** 2025-02-05

**Authors:** Ryosuke Sato, Kazuyuki Matsumoto, Mayu Uka, Kosei Takagi, Kenji Nishida, Takehiro Tanaka, Yuki Fujii, Koichiro Tsutsumi, Shigeru Horiguchi, Motoyuki Otsuka

**Affiliations:** 1 Department of Gastroenterology and Hepatology, Okayama University Hospital, 2-5-1 Shikata-cho, Kita-ku, Okayama, Okayama 700-8558 Japan; 2 Department of Radiology, Okayama University Graduate School of Medicine, Dentistry, and Pharmaceutical Sciences, 2-5-1 Shikata-cho, Kita-ku, Okayama, Okayama 700-8558 Japan; 3 Department of Gastroenterological Surgery, Okayama University Graduate School of Medicine, Dentistry, and Pharmaceutical Sciences, 2-5-1 Shikata-cho, Kita-ku, Okayama, Okayama 700-8558 Japan; 4 Department of Pathology, Dentistry and Pharmaceutical Science, Okayama University Graduate School of Medicine, 2-5-1 Shikata-cho, Kita-ku, Okayama, Okayama 700-8558 Japan

**Keywords:** Pancreatic intraductal neoplasms, Pancreatic carcinoma, Intraductal tubulopapillary neoplasm, Genetic testing

## Abstract

We herein report a case of pancreatic ductal adenocarcinoma (PDAC) that developed within the pancreatic duct and was initially diagnosed as an intraductal tubulopapillary neoplasm (ITPN). A 76-year-old man presented with weight loss and main pancreatic duct dilation. The imaging studies revealed a 30-mm hypovascular tumor within the main duct of the pancreatic head. An endoscopic examination with a biopsy revealed high-grade atypical epithelial cells with immunostaining patterns suggestive of ITPN. Following robot-assisted pancreaticoduodenectomy, postoperative pathology revealed conflicting features: nodular/cribriform infiltrations typical of ITPN and non-lobular replacement with scattered infiltrations characteristic of PDAC. A comprehensive genomic profiling test detected *KRAS* and *TP53* mutations, leading to the final diagnosis of PDAC (fT3N1aM0, stage IIB). The patient received adjuvant S-1 chemotherapy and remained recurrence-free for 15 months post-surgery. This case highlights the diagnostic challenges of differentiating intraductal pancreatic tumors and demonstrates the utility of integrating genetic testing with conventional diagnostic modalities for an accurate diagnosis and appropriate treatment selection.

**Supplementary Information:**

The online version contains supplementary material available at 10.1007/s12328-025-02098-y.

## Introduction

Pancreatic tumors exhibit diverse growth patterns and invasion characteristics, resulting in distinct imaging features for each type [1]. Intraductal papillary mucinous neoplasm (IPMN) and intraductal tubulopapillary neoplasm (ITPN) are well-known entities classified as intraductal neoplasms. Other tumors, such as acinar cell carcinoma, neuroendocrine neoplasms, and rarely, pancreatic ductal adenocarcinoma (PDAC), can also develop within the pancreatic duct [2]. The similarity in imaging characteristics and challenges associated with obtaining biopsy specimens from the pancreatic duct often complicate a preoperative differential diagnosis, sometimes extending the diagnostic uncertainty to a histopathological examination.

Accurate differentiation between PDAC and other pancreatic tumors is crucial for determining appropriate treatment strategies and predicting prognoses. PDAC is the only pancreatic tumor with established evidence supporting the use of postoperative adjuvant chemotherapy [3]. However, such evidence is lacking for other pancreatic tumor types. Consequently, a precise diagnosis is paramount when considering postoperative adjuvant chemotherapy.

Recent studies have demonstrated the utility of genetic testing in cases where conventional imaging, pathological examinations, and immunostaining prove insufficient for differentiating between IPMN, ITPN, and PDAC [4]. PDAC exhibits distinct genetic mutations, with *KRAS* mutations present in approximately 95% of cases and *TP53* mutations in about 75%. In contrast, ITPN shows different genetic changes from PDAC, including *FGFR2* fusions and *PIK3CA* mutations [5–7]. These genetic differences can serve as additional diagnostic tools when conventional imaging and pathological examinations yield inconclusive results.

We herein report a case of a pancreatic tumor growing within the pancreatic duct that was initially preoperatively diagnosed as ITPN. However, postoperative immunohistochemistry and genetic testing led to the final diagnosis of PDAC, highlighting the importance of comprehensive diagnostic approaches in challenging cases.

## Case report

A 76-year-old man was admitted with a suspected pancreatic tumor following a 7-kg weight loss over 6 months and main pancreatic duct dilation on abdominal computed tomography (CT). His medical history included surgery for descending colon cancer 30 years prior and an iodine allergy but no pancreatic disease or family history of malignancy. The patient had no history of alcohol consumption or smoking. A physical examination revealed no abdominal tenderness or jaundice. Laboratory investigations revealed an elevated serum carbohydrate antigen (CA)19–9 level of 117.1 U/mL and duke pancreatic monoclonal antigen type 2 (DUPAN-2) of 236 U/mL, while other laboratory data, including complete blood count, and liver and renal function tests, were within normal limits (Table S1).

**Fig. 1:**
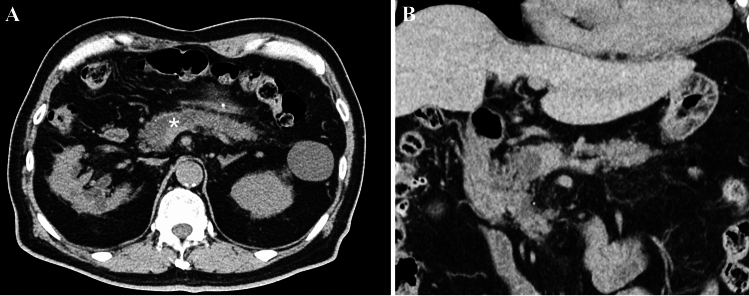
Axial (**A**) and coronal (**B**) images of the plain abdominal computed tomography (CT) findings. CT showed main pancreatic duct dilation (asterisk), and the pancreatic parenchyma showed mild atrophy in the body and tail, but the pancreatic mass was difficult to confirm

**Fig. 2:**
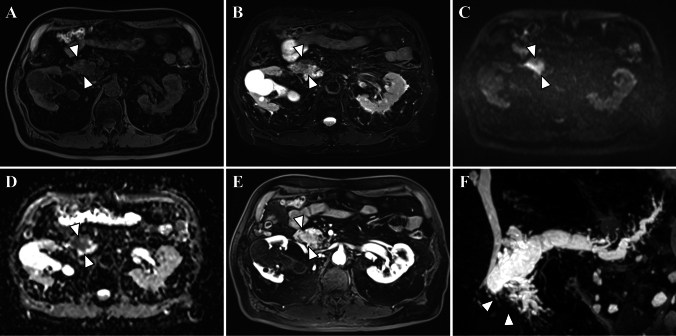
Magnetic resonance imaging (MRI) findings. MRI revealed a mass in the main pancreatic duct of the pancreatic head (arrowhead). The mass was recognized as a low-intensity area on T1-weighted imaging (T1WI) (**A**) and T2WI (**B**). It was hyperintense on diffusion-weighted images (DWI) (b = 800 s/mm^2^) (**C**) with decreased apparent diffusion coefficient (ADC) values (**D**). **E** The mass was not well contrasted on gadolinium-enhanced MRI (Gd-MRI). **F** On magnetic resonance cholangiopancreatography (MRCP), the main pancreatic duct was obstructed at the head of the pancreas (arrowhead). The dilation of the branch pancreatic ducts tail side to the obstruction was observed

**Fig. 3:**
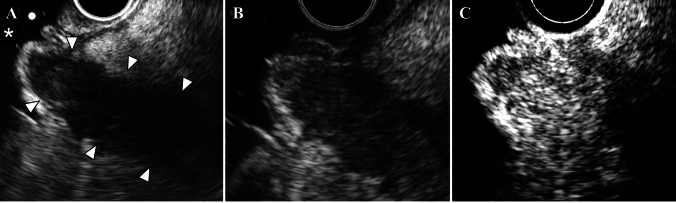
Endoscopic ultrasonography (EUS) findings of the duodenum lumen (dot), major papilla (asterisk), and the pancreatic head observed from the second portion of the duodenum. **A**, **B** EUS displayed a hypoechoic mass completely filling and conforming to the shape of the dilated main pancreatic duct (arrowhead). **C** On contrast-enhanced EUS, the mass showed a relatively homogeneous and delayed contrast effect

**Fig. 4:**
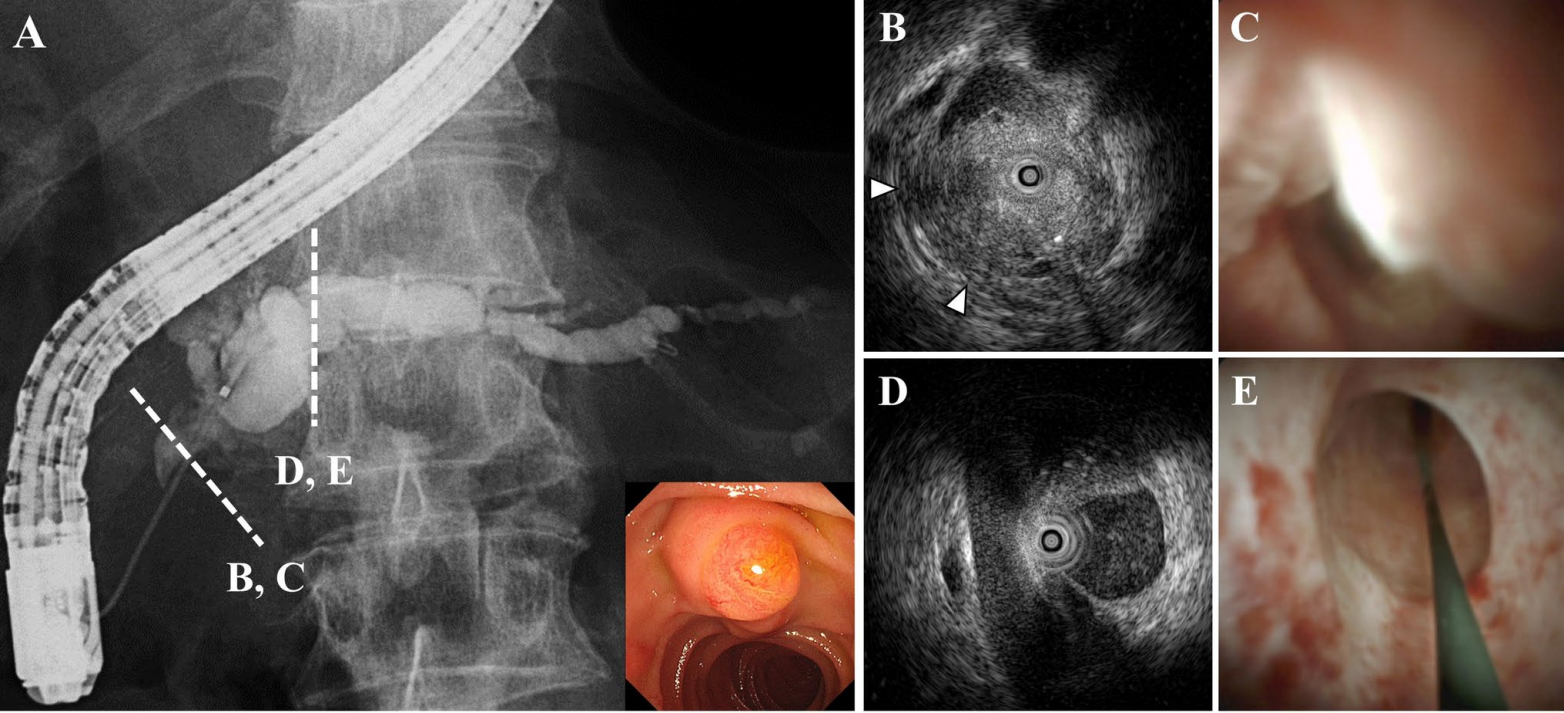
Endoscopic retrograde cholangiopancreatography (ERCP), intraductal ultrasonography (IDUS), and peroral pancreatoscopy (POPS, SpyScope DSII; Boston Scientific, Massachusetts, USA) findings. **A** ERCP revealed a defect in the main pancreatic duct at the head of the pancreas and dilation of the tail-side main pancreatic duct. **B**, **C** IDUS and POPS revealed an intraductal tumor filling the main pancreatic duct and invasion into the pancreatic parenchyma was suspected (arrowhead). **D**, **E** The main pancreatic duct in the body of the pancreas was intact, and no masses were observed

While plain abdominal CT showed dilation of the main pancreatic duct and atrophy of the pancreatic parenchyma in the body and tail, the pancreatic mass was difficult to confirm (Fig. 1). Gadolinium-enhanced magnetic resonance imaging (Gd-MRI) revealed a 30-mm hypovascular tumor in the main duct of the pancreatic head, with dilation of the main and branch pancreatic ducts (Fig. 2A–F). Endoscopic ultrasonography (EUS) revealed a hypoechoic mass with a contrast effect completely filling and conforming to the shape of the dilated main pancreatic duct (Fig. 3A, B). Endoscopic retrograde cholangiopancreatography (ERCP), intraductal ultrasonography (IDUS), and peroral pancreatoscopy (POPS, SpyScope DSII; Boston Scientific, Massachusetts, USA) confirmed main pancreatic duct dilation and revealed an intraductal tumor inside the main pancreatic duct of the pancreatic head (Fig. 4A–E).

**Fig. 5:**
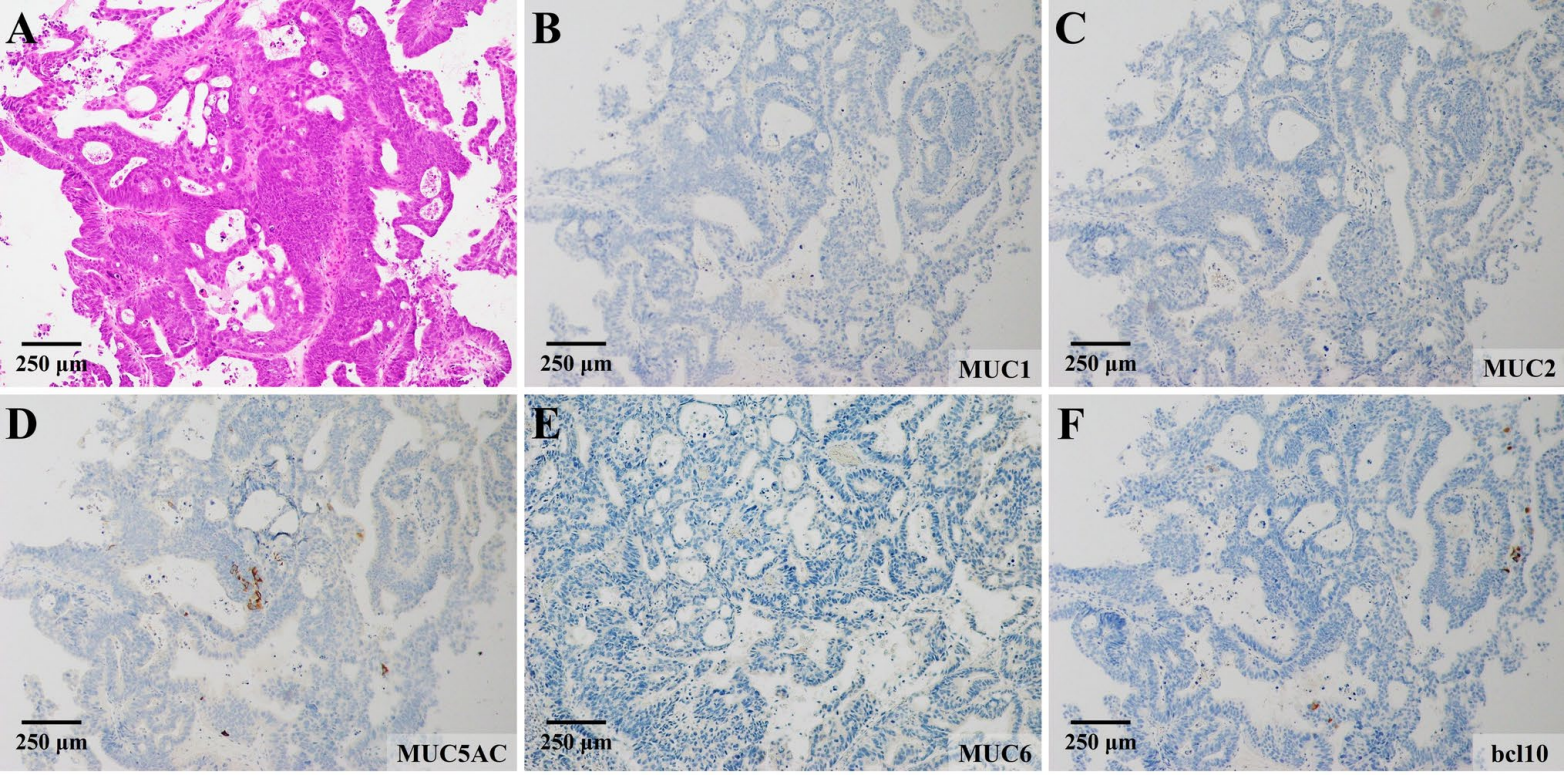
Histological findings obtained from the biopsies via POPS. **A** High-grade atypical epithelial cells proliferating in a fused ductal pattern were observed by a biopsy via POPS. Immunohistochemistry results were MUC1-negative (**B**), MUC2-negative (**C**), MUC5AC-negative (**D**), partially MUC6-positive (**E**), and bcl10-negative (**F**)

A tumor biopsy via POPS showed high-grade atypical epithelial cells proliferating in a fused ductal pattern (Fig. 5A). The immunohistochemistry results were MUC1-negative, MUC2-negative, MUC5AC-negative, partially MUC6-positive, and bcl10-negative (Fig. 5B–F). These findings, along with the absence of mucus production, ruled out intraductal papillary mucinous neoplasm (IPMN) and pancreatic acinar cell carcinoma. Based on the tumor’s location within the pancreatic duct, it was preoperatively diagnosed as intraductal tubulopapillary neoplasm (ITPN) (cT2N0M0, stage IB), and robot-assisted pancreaticoduodenectomy was performed.

**Fig. 6:**
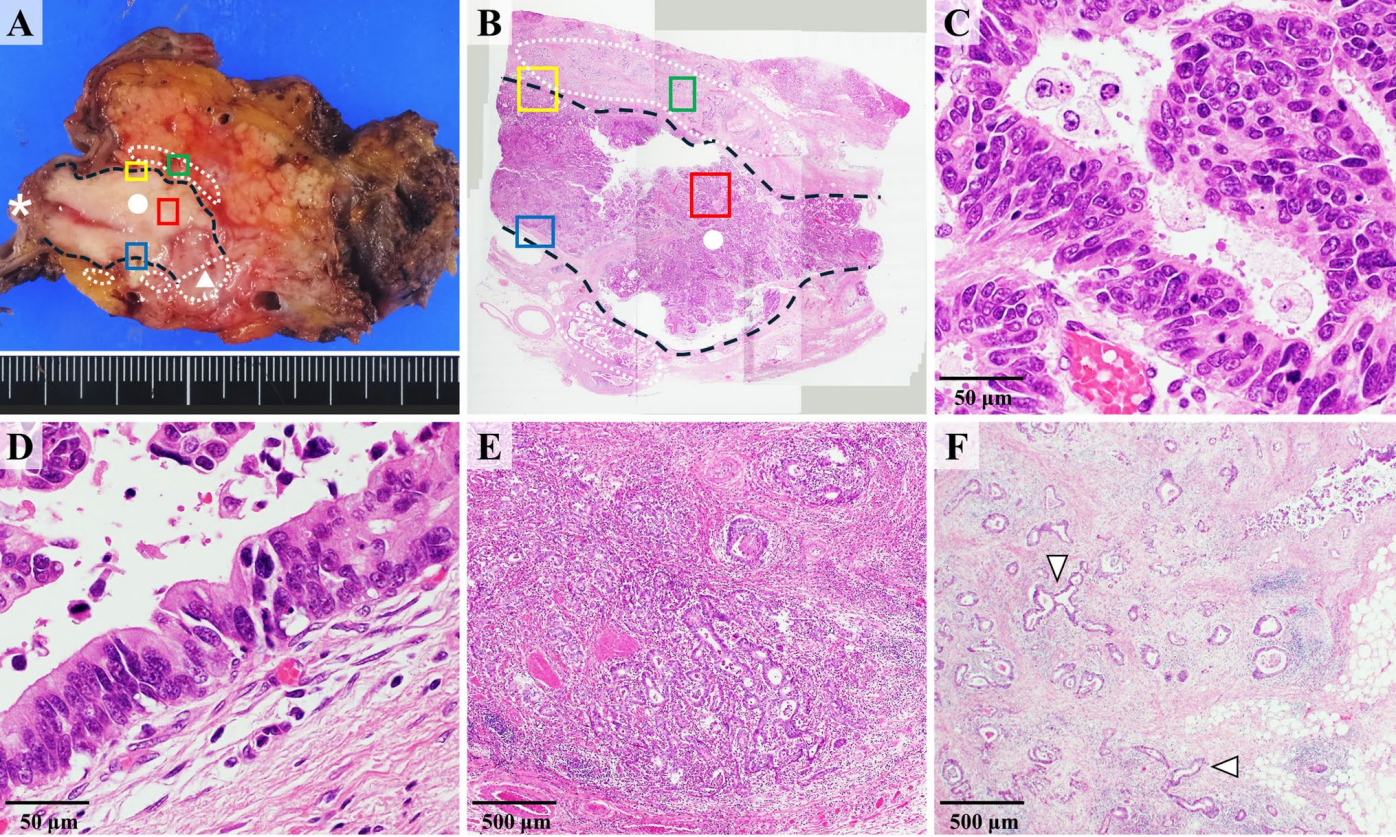
Histological findings obtained from the surgical specimen. **A**, **B** Postoperative macroscopic pathology revealed a tumor filling the main pancreatic duct (dot and black broken line) continuing from the major papilla (asterisk) and also showed invasion into the pancreatic parenchyma (triangle). The wall of the main pancreatic duct was irregular due to the replacement of tumor cells, and the demarcation between the wall and the tumor was unclear in the invaded areas. Infiltrative lesions beyond the main pancreatic duct were also observed (white broken line). **C** An infiltrative adenocarcinoma growing in tubular and cribriform patterns within the main pancreatic duct (red square). **D** High-grade atypical epithelial cells were also observed lining the main pancreatic duct (blue square). **E** The nodular and cribriform infiltrations like the pancreatic duct lesions were initially considered consistent with invasive intraductal tubulopapillary neoplasm (ITPN) (yellow square). **F** Meanwhile, the presence of non-lobular replacement and scattered infiltrations in the pancreatic parenchyma (arrowhead) were findings more reminiscent of PDAC (green square)

A postoperative macroscopic pathological examination revealed a tumor filling the main pancreatic duct and infiltrating lesions beyond the main pancreatic duct (Fig. 6A, B). A microscopic examination showed an infiltrative adenocarcinoma growing in tubular and cribriform patterns within the main pancreatic duct (Fig. 6C). High-grade atypical epithelial cells were also observed lining the main pancreatic duct (Fig. 6D). The immunohistochemistry results were consistent with those of the preoperative biopsy. The nodular and cribriform infiltrations, similar to the lesions in the pancreatic duct, were initially considered consistent with invasive ITPN (Fig. 6E). However, the presence of non-lobular replacement and scattered infiltrations beyond the main pancreatic duct was more reminiscent of PDAC than ITPN (Fig. 6F). These conflicting histological features posed a significant challenge in determining the precise histological type based on the pathology alone.

At the patient’s request and expense, a cancer gene panel test (FoundationOne; Foundation Medicine, Massachusetts, USA) using a surgical specimen was performed, revealing KRAS and TP53 mutations, which are frequently seen in PDAC, but no genetic abnormalities such as MCL, FGFR2, and PIK3CA were typically associated with ITPN [4]. Based on a comprehensive assessment of the pathology results and genetic testing, the patient was diagnosed with invasive PDAC (fT3N1aM0, stage IIB). The patient received S-1 as postoperative adjuvant chemotherapy for 6 months and remained recurrence-free for 15 months post-surgery.

## Discussion

Pancreatic tumors that develop and grow within the pancreatic ducts include ITPN, IPMN, PDAC, neuroendocrine neoplasm (NEN), and acinar cell carcinoma (ACC) [2]. While these tumors have distinct characteristics, their similar growth patterns within the pancreatic duct can make differentiation challenging through conventional diagnostic approaches. ITPN, classified as a pancreatic intraductal neoplasm in the 2019 WHO tumor classification, is a rare solid nodular tumor obstructing dilated pancreatic ducts, lacking mucin, and composed of uniformly high-grade epithelial cells [8].

The differentiation between ITPN and PDAC is sometimes particularly challenging, as both can present as solid tumors with similar enhancement patterns in imaging studies [9]. In our case, despite comprehensive imaging studies and immunohistochemical analysis of the biopsy specimen, a definitive preoperative diagnosis remained elusive. The postoperative pathological examination revealed features characteristic of both ITPN and PDAC, necessitating additional diagnostic approaches.

The genetic profile of pancreatic tumors has emerged as a crucial differentiating factor in such challenging cases. Table 1 shows the comparison of gene alterations in PDAC and ITPN, and the results for this case. The genetic mutations observed in our case—notably *KRAS* and *TP53* mutations—strongly aligned with the characteristic mutation pattern of PDAC. These mutations, present in approximately 95% and 75% of PDAC cases respectively, are rarely observed in ITPN. Conversely, genetic changes typically associated with ITPN, such as *MCL* amplifications (31.8%), *FGFR2* fusions (18.2%), and *PIK3CA* mutations (13.6%), were absent in our case [5–7]. Recent comprehensive analysis has shown that these distinct genetic profiles reflect fundamentally different oncogenic pathways between PDAC and ITPN. While PDAC is characterized by *KRAS*-dependent signaling activation and loss of tumor suppressor function through *TP53* mutations, ITPN shows enrichment of alterations affecting growth factor signaling pathways through *FGFR2* fusions and *PI3K* pathway activation. Furthermore, molecular pathway analysis has demonstrated that *PI3K-Akt* signaling is the most activated pathway in ITPN, suggesting potential therapeutic implications [5]. In our case, genetic testing proved particularly valuable as conventional histopathological and immunohistochemical findings were inconclusive. The molecular profile definitively supported the diagnosis of PDAC, enabling us to implement appropriate adjuvant chemotherapy based on established evidence. This case highlights the critical role that comprehensive genetic analysis can play in resolving diagnostically challenging cases, ultimately leading to optimal treatment selection.

**Table 1:** Comparison of gene alterations in PDAC and ITPN, and the results for this case [5–7]

Altered gene	Frequency in PDAC (%)	Frequency in ITPN (%)	Common pattern	This case
*KRAS*	93.0	10.4	Missense	Positive
*TP53*	72.0	4.7	Missense	Positive
*CDKN2A*	30.0	27.2	Missense Truncating Deletion	Negative
*SMAD4*	32.0	0	Missense Truncating Deletion	Negative
*MCL*	0.1	31.8	Amplifications	Negative
*FGFR2*	0.2	18.2	Fusions	Negative
*PIK3CA*	2.1	13.6	Missense	Negative

*PDAC* pancreatic ductal adenocarcinoma, *ITPN* intraductal tubulopapillary neoplasm

An intraductal growth pattern called “cancerization of ducts (COD)” is reported in approximately 60% of surgically resected PDAC cases [10]. Recent studies have shown that COD is associated with female gender, advanced T stage, lymphovascular invasion, perineural invasion, and R1 resection margin status. Notably, it does not significantly impact overall survival or recurrence-free survival [11]. These findings suggest that COD status may have implications for treatment planning, particularly in the preoperative setting.

The treatment approach for resectable PDAC has been well-established, with proven efficacy of neoadjuvant chemotherapy using gemcitabine and S-1 combination therapy [12] and adjuvant chemotherapy with either gemcitabine monotherapy [13, 14] or S-1 monotherapy [3]. In contrast, rare pancreatic tumors such as ITPN lack established evidence for pre- or post-operative chemotherapy, necessitating careful case-by-case consideration. In our patient, although the preoperative diagnosis of PDAC was not possible, precluding neoadjuvant chemotherapy, the postoperative diagnosis of PDAC through genetic testing enabled the implementation of evidence-based adjuvant chemotherapy. The patient’s recurrence-free survival of 15 months to date may be partly attributed to this accurate postoperative diagnosis and subsequent appropriate adjuvant therapy. This case highlights the critical impact that an accurate diagnosis can have on treatment decisions and potentially on patient outcomes.

This case emphasizes the importance of a multifaceted diagnostic approach for pancreatic tumors, particularly when conventional imaging and postoperative pathology yield inconclusive results. The integration of genetic testing can play a crucial role in confirming the diagnosis and guiding treatment decisions, particularly regarding chemotherapy. However, it is essential that no single diagnostic modality be relied upon exclusively. A comprehensive evaluation incorporating imaging, pathological findings, and genetic testing results is necessary for an accurate diagnosis and optimal treatment planning.

Given the rarity of intraductal PDAC and ITPN, a further case accumulation and analysis are vital to establish robust evidence for future treatment strategies and enhance our understanding of these challenging pancreatic neoplasms. Future research should focus on developing more specific imaging criteria to differentiate pancreatic intraductal tumors and identify novel biomarkers for an accurate diagnosis. In addition, long-term follow-up studies of cases such as ours could provide valuable insights into the natural history and prognosis of intraductal PDAC misdiagnosed as ITPN.

In conclusion, we experienced a case in which we initially diagnosed ITPN based on preoperative imaging tests and finally diagnosed PDAC based on a detailed pathological examination and genetic testing and were able to perform appropriate postoperative adjuvant chemotherapy.

## Supplementary Information

Below is the link to the electronic supplementary material.

Supplementary file1 (DOCX 22 KB)
