# Supplementary material for: A case of pancreatic ductal adenocarcinoma growing within the pancreatic duct mimicking an intraductal tubulopapillary neoplasm
Source: Clin J Gastroenterol. 2025 Feb 5;18(2):376–82. doi: 10.1007/s12328-025-02098-y (PMC11922994; doi:10.1007/s12328-025-02098-y)
Supplement: Supplementary file 1 — Supplementary file1 (DOCX 22 KB) [file 12328_2025_2098_MOESM1_ESM.docx]

**Table S1.** Laboratory data (reference range, units)

| < Hematology > | | | < Biochemistry > | | | < Tumor marker > | | |
| --- | --- | --- | --- | --- | --- | --- | --- | --- |
| WBC | 7550 | (3300-8600, /μL) | TP | 7.3 | (6.6-8.1, g/dL) | CEA | 2.68 | (<5.00, ng/mL) |
| Neu | 65.1 | (40.0-70.0, %) | Alb | 4.1 | (4.1-5.1, g/dL) | CA19-9 | 117.1 | (0.0-35.4, U/mL) |
| Ly | 25.8 | (16.5-49.5, %) | T.Bil | 0.47 | (0.40-1.50, mg/dL) | DUPAN-2 | 236 | (0-150, U/mL) |
| RBC | 471 | (435-555, ×10^4^/μL) | AST | 31 | (13-30, U/L) | SPan-1 | 35.6 | (0.0-30.0, U/mL) |
| Hb | 15.1 | (13.7-16.8, g/dL) | ALT | 36 | (10-42, U/L) |  |  |  |
| Ht | 46.2 | (40.7-50.1, %) | γ-GTP | 33 | (38-75, U/L) |  | | |
| PLT | 16.9 | (15.8-34.8, ×10^4^/μL) | ALP | 73 | (38-113, U/L) |  |  |  |
|  |  |  | AMY | 61 | (44-132, U/L) |  |  |  |
| < Coagulation > | | | LIPA | 119 | (13-55, U/L) |  |  |  |
| APTT | 32.5 | (26.9-38.1, sec) | BUN | 15.9 | (8.0-20.0,mg/dL) |  |  |  |
| PT | 87 | (73-118, %) | CRE | 0.91 | (0.65-1.07, mg/dL) |  |  |  |
| PT-INR | 1.07 | (<2.99) | Na | 138 | (138-145, mmol/L) |  |  |  |
| D-dimer | 0.5 | (0.0-0.9, μg/mL) | K | 4.2 | (3.6-4.8, mmol/L) |  |  |  |
|  |  |  | Cl | 106 | (101-108, mmol/L) |  |  |  |
|  |  |  | Ca | 8.9 | (8.8-10.1, mg/dL) |  |  |  |
|  |  |  | CRP | 0.06 | (<0.15, mg/dL) |  |  |  |

CEA, Carcinoembryonic antigen; CA19-9, Carbohydrate antigen 19-9; DUPAN-2, Duke pancreatic monoclonal antigen type 2; SPan-1, Serum s-pancreas-1 antigen
